# Supplementary material for: Intake of Boiled Potato in Relation to Cardiovascular Disease Risk Factors in a Large Norwegian Cohort: The HUNT Study
Source: Nutrients. 2019 Dec 27;12(1):73. doi: 10.3390/nu12010073 (PMC7019529; doi:10.3390/nu12010073)
Supplement: Supplementary file 1 [file nutrients-12-00073-s001.pdf]

Table S1. Odds ratio (OR) for adverse levels of components of the metabolic syndrome and cardiovascular disease (CVD) risk factors according to potato consumption.

CVD Risk Factor and Frequency of Boiled Potato Consumption	Number of Cases	Number of Non-Cases	Crude OR	Adjusted OR [a]	95% CI
Body mass index, kg/m^2					
<1 per week	1400	1138	1.00	1.00	Reference
1–3 per week	7363	4615	1.30	1.12	1.02–1.22
4–6 per week	12,110	5671	1.74	1.19	1.08–1.30
≥1 per day	8077	3309	1.98	1.10	0.99–1.21
Waist circumference, >88 cm for women/ >102 cm for men					
<1 per week	812	1710	1.00	1.00	Reference
1–3 per week	4215	7702	1.15	1.10	0.99–1.20
4–6 per week	7283	10447	1.47	1.20	1.09–1.33
≥1 per day	5251	6115	1.81	1.16	1.05–1.29
Hypertension, 130/85 mmHg					
<1 per week	824	1706	1.00	1.00	Reference
1–3 per week	4591	7348	1.29	0.96	0.86–1.10
4–6 per week	9157	8578	2.21	0.96	0.87–1.07
≥1 per day	7485	3859	4.02	1.04	0.93–1.16
Total cholesterol, ≥6 mmol/L					
<1 per week	604	1934	1.00	1.00	Reference
1–3 per week	3369	8609	1.25	1.04	0.94–1.16
4–6 per week	6486	11,295	1.84	1.13	1.02–1.25
≥1 per day	4933	6453	2.45	1.12	1.01–1.25
Triglycerides, ≥1.7 mmol/L					
<1 per week	715	1775	1.00	1.00	Reference
1–3 per week	3799	8021	1.18	1.09	0.98–1.21
4–6 per week	6552	11,057	1.47	1.19	1.07–1.32
≥1 per day	4569	6695	1.69	1.20	1.07–1.34
HDL, <1.29 mmol/L for women/ <1.03 mmol/L for men					
<1 per week	740	1699	1.00	1.00	Reference
1–3 per week	3767	7861	1.10	1.17	1.03–1.32
4–6 per week	5628	11,734	1.10	1.20	1.06–1.36
≥1 per day	3586	7540	1.09	1.30	1.14–1.48
Metabolic syndrome [b]					
<1 per week	411	2007	1.00	1.00	Reference
1–3 per week	2272	9269	1.20	1.07	0.95–1.21
4–6 per week	4452	12,821	1.70	1.19	1.05–1.34
≥1 per day	3475	7603	2.23	1.17	1.03–1.33

Abbreviations: HDL = high-density lipoprotein. [a] Adjusted for age (continuous), sex (woman, man), cardiovascular disease (no, yes), work type (mostly sedentary, much walking, much lifting, heavy work, unknown/not employed), frequency of physical activity (none, <1, 1, 2–3, ≥4 times per week), smoking (never, former, current), intake of alcohol (≤1 month, ≤1 per week, ≥2 per week, abstainer), intake of vegetables (≤3 times per week, 4–6 times per week, ≥1 times per day), intake of fish (≤3 times per month, 1–3 times per week, ≥4 times per week), intake of processed meat (≤3 times per month,1–3 times per week, ≥4 times per week), intake of pasta/rice (≤3 times per month, 1–3 times per week, ≥4 times per week). All outcomes except body mass index and waist circumference were adjusted also for body mass index. [b] Metabolic syndrome is defined as having adverse levels of at least three of these variables: high waist circumference, hypertension, high triglycerides, low HDL cholesterol.

Table S2. Odds ratio (OR) for adverse levels of components of the metabolic syndrome and cardiovascular disease risk factors according to potato consumption, stratified by sex.

Women	Men

CVD Risk Factor and Frequency of Boiled Potato Consumption	Number of Cases	Adjusted OR [a]	95% CI	Number of Cases	Adjusted OR [a]	95% CI
Body mass index, kg/m^2						
<1 per week	1537	1.00	Reference	1001	1.00	Reference
1–3 per week	6912	1.08	0.96–1.22	5066	1.13	0.98–1.32
4–6 per week	9630	1.20	1.07–1.35	8151	1.14	0.98–1.32
≥1 per day	6190	1.10	0.97–1.25	5196	1.04	0.88–1.23
Waist circumference, >88 cm for women/ >102 cm for men						
<1 per week	1522	1.00	Reference	1000	1.00	Reference
1–3 per week	6852	1.11	0.99–1.25	5065	1.00	0.84–1.18
4–6 per week	9585	1.26	1.11–1.42	8145	1.06	0.89–1.25
≥1 per day	6178	1.16	1.01–1.32	5188	1.09	0.91–1.30
Hypertension, 130/85 mmHg						
<1 per week	1531	1.00	Reference	999	1.00	Reference
1–3 per week	6889	0.96	0.83–1.12	5050	0.95	0.82–1.11
4–6 per week	9605	0.99	0.85–1.15	8130	0.97	0.83–1.13
≥1 per day	6162	1.11	0.94–1.30	5182	0.99	0.84–1.17
Total cholesterol, ≥6 mmol/L						
<1 per week	1537	1.00	Reference	1001	1.00	Reference
1–3 per week	6912	0.95	0.83–1.09	5066	1.16	0.99–1.37
4–6 per week	9630	1.00	0.87–1.14	8151	1.34	1.14–1.58
≥1 per day	6190	1.02	0.88–1.17	5196	1.31	1.10–1.55
Triglycerides, ≥1.7 mmol/L						
<1 per week	1503	1.00	Reference	987	1.00	Reference
1–3 per week	6809	0.90	0.77–1.04	5011	1.31	1.13–1.53
4–6 per week	9538	1.03	0.89–1.19	8071	1.41	1.21–1.64
≥1 per day	6109	1.03	0.88–1.20	5155	1.42	1.21–1.67
HDL, <1.29 mmol/L for women/ <1.03 mmol/L for men						
<1 per week	1470	1.00	Reference	969	1.00	Reference
1–3 per week	6692	1.17	0.95–1.44	4936	1.15	0.98–1.34
4–6 per week	9383	1.33	1.08–1.64	7979	1.13	0.97–1.32
≥1 per day	6036	1.48	1.18–1.85	5090	1.20	1.01–1.42
Metabolic syndrome [b]						
<1 per week	1451	1.00	Reference	967	1.00	Reference
1–3 per week	6617	1.06	0.87–1.29	4924	1.07	0.91–1.26
4–6 per week	9320	1.26	1.04–1.52	7953	1.16	0.99–1.36
≥1 per day	6004	1.22	1.00–1.48	5074	1.14	0.96–1.35

Abbreviations: OR = Odds ratio, CI = Confidence interval, HDL = high-density lipoprotein cholesterol. [a] Adjusted for age (continuous), cardiovascular disease (no, yes), work type (mostly sedentary, much walking, much lifting, heavy work, unknown/not employed), frequency of physical activity (none, <1, 1, 2–3, ≥4 times per week), smoking (never, former, current), intake of alcohol (≤1 month, ≤1 per week, ≥2 per week, abstainer), intake of vegetables (≤3 times per week, 4–6 times per week, ≥1 times per day), intake of fish (≤3 times per month, 1–3 times per week, ≥4 times per week), intake of processed meat (≤3 times per month, 1–3 times per week, ≥4 times per week), intake of pasta/rice (≤3 times per month, 1–3 times per week, ≥4 times per week). All outcomes except body mass index and waist circumference were adjusted also for body mass index. [b] Metabolic syndrome is defined as having adverse levels of at least three of these variables: high waist circumference, hypertension, high triglycerides, low HDL cholesterol.

Table S3. Odds ratio (OR) for adverse levels of components of the metabolic syndrome and cardiovascular disease risk factors according to potato consumption, stratified by body mass index.

	<25 kg/m²			≥25 kg/m²		
**CVD Risk Factor and Frequency of Boiled Potato Consumption**	**Number of Cases**	**Adjusted OR [a]**	**95% CI**	**Number of Cases**	**Adjusted OR [a]**	**95% CI**
Waist circumference, >88 cm for women/ >102 cm for men						
<1 per week	1134	1.00	Reference	1388	1.00	Reference
1–3 per week	4603	0.99	0.74–1.31	7314	1.03	0.90–1.17
4–6 per week	5657	1.04	0.78–1.39	12,073	1.12	0.98–1.28
≥1 per day	3306	0.89	0.65–1.22	8060	1.17	1.02–1.34
Hypertension, 130/85 mmHg						
<1 per week	1137	1.00	Reference	1393	1.00	Reference
1–3 per week	4596	0.95	0.79–1.15	7343	0.95	0.79–1.15
4–6 per week	5652	0.96	0.80–1.15	12,083	0.96	0.80–1.15
≥1 per day	3299	1.11	0.91–1.35	8045	1.11	0.91–1.35
Total cholesterol, ≥6 mmol/L						
<1 per week	1138	1.00	Reference	1400	1.00	Reference
1–3 per week	4615	1.02	0.85–1.23	7363	1.02	0.90–1.16
4–6 per week	5671	1.08	0.90–1.30	12,110	1.11	0.98–1.26
≥1 per day	3309	1.08	0.89–1.32	8077	1.11	0.97–1.27
Triglycerides, ≥1.7 mmol/L						
<1 per week	1114	1.00	Reference	1376	1.00	Reference
1–3 per week	4551	0.96	0.80–1.17	7269	1.13	1.00–1.29
4–6 per week	5612	1.02	0.84–1.24	11,997	1.25	1.10–1.41
≥1 per day	3291	1.16	0.93–1.43	7973	1.22	1.07–1.39
HDL, <1.29 mmol/L for women/ <1.03 mmol/L for men						
<1 per week	1087	1.00	Reference	1352	1.00	Reference
1–3 per week	4476	1.32	1.07–1.63	7152	1.09	0.94–1.27
4–6 per week	5524	1.31	1.05–1.63	11,838	1.14	0.98–1.33
≥1 per day	3260	1.44	1.13–1.83	7866	1.23	1.05–1.44
Metabolic syndrome [b]						
<1 per week	1081	1.00	Reference	1337	1.00	Reference
1–3 per week	4451	1.29	0.82–2.02	7090	1.00	0.87–1.14
4–6 per week	5495	1.33	0.91–2.23	11,778	1.09	0.96–1.25
≥1 per day	3246	1.45	0.91–2.33	7832	1.11	0.96–1.27

Abbreviations: OR = Odds ratio, CI = Confidence interval, HDL = high-density lipoprotein cholesterol. [a] Adjusted for age (continuous), sex (woman, man), cardiovascular disease (no, yes), work type (mostly sedentary, much walking, much lifting, heavy work, unknown/not employed), frequency of physical activity (none, <1, 1, 2–3, ≥4 times per week), smoking (never, former, current), intake of alcohol (≤1 month, ≤1 per week, ≥2 per week, abstainer), intake of vegetables (≤3 times per week, 4–6 times per week, ≥1 times per day), intake of fish (≤3 times per month, 1–3 times per week, ≥4 times per week), intake of processed meat (≤3 times per month, 1–3 times per week, ≥4 times per week), intake of pasta/rice (≤3 times per month, 1–3 times per week, ≥4 times per week). All outcomes except waist circumference were adjusted also for body mass index. [b] Metabolic syndrome is defined as having adverse levels of at least three of these variables: high waist circumference, hypertension, high triglycerides, low HDL cholesterol.
